# Integrated transcriptomic and regulatory network analyses uncovers the role of let-7b-5p, SPIB, and HLA-DPB1 in sepsis

**DOI:** 10.1038/s41598-022-16183-6

**Published:** 2022-07-13

**Authors:** Mohd Mohsin, Prithvi Singh, Salman Khan, Amit Kumar Verma, Rishabh Jha, Mohammed A. Alsahli, Arshad Husain Rahmani, Saleh A. Almatroodi, Faris Alrumaihi, Nisha Kaprwan, Kapil Dev, Ravins Dohare, Mansoor Ali Syed

**Affiliations:** 1 Department of Biotechnology, Faculty of Natural Sciences, Jamia Millia Islamia, New Delhi, 110025 India; 2 Centre for Interdisciplinary Research in Basic Sciences, Jamia Millia Islamia, New Delhi, 110025 India; 3 Department of Medical Laboratories, College of Applied Medical Sciences, Qassim University, Buraydah, 51452 Saudi Arabia; 4 Department of Computer Science, Faculty of Natural Sciences, Jamia Millia Islamia, New Delhi, 110025 India

**Keywords:** Computational biology and bioinformatics, Genetics, Systems biology

## Abstract

Sepsis has affected millions of populations of all age groups, locations, and sexes worldwide. Immune systems, either innate or adaptive are dysregulated due to the infection. Various biomarkers are present to date, still sepsis is a primary cause of mortality. Globally, post-operative body infections can cause sepsis and septic shock in ICU. Abnormal antigen presentation to T-cells leads to a dysregulated immune system. miRNAs are sparkly evolved as biomarkers due to their high sensitivity and efficiency. In this work, we analyzed high-throughput mRNA data collected from Gene Expression Omnibus (GEO) and linked it to significant miRNAs and TFs using a network-based approach. Protein–protein interaction (PPI) network was constructed using sepsis-specific differentially expressed genes (DEGs) followed by enrichment analyses and hub module detection. Sepsis-linked decrease transcription of the classical HLA gene such as HLA-DPB1 and its interplay with miR-let-7b-5p and transcription factor SPIB was observed. This study helped to provide innovative targets for sepsis.

## Introduction

Sepsis is described as life-threatening organ dysfunction provoked by a dysregulated host response to infection. The infection can be induced by bacteria, fungi, viruses, and parasitic pathogens. Sepsis is the primary cause of morbidity and mortality^1^. Although the global concern of sepsis is challenging to anticipate, The Global Burden of Diseases (GBD) 2017 has evaluated the universal, territorial, and nationwide proportion of sepsis, and they also estimated the mortality from this ailment across 195 countries and territories. Around 48.9 million cases of sepsis were reported, and 11 million sepsis-related deaths in 2017 were recorded worldwide. From 1990 to 2017, the primary reason for sepsis was diarrhoeal disease in all age groups, locations, and both sexes, especially in children younger than 5 years. But worldwide, the primary inducer of sepsis-related deaths was a lower respiratory infection, with 1.8 million deaths in 2017. Moreover, the sepsis-related deaths in children younger than 5 years old are neonatal disorders, lower respiratory infections, and diarrhoeal diseases^2^.

The harshness of organ dysfunction can be estimated by the SOFA (Sequential [Sepsis-Related] Organ Failure Assessment) score for a better clinical understanding. More than or equal to 2 points of SOFA score represents organ dysfunction and increases the mortality chances of approximately 10% with surmise infection in a hospital population. The score can be assessed by specific biomarkers such as respiration rate, coagulation, bilirubin, blood pressure, creatinine, and urine output. The standard SOFA score was marked to be zero except for the patients who had pre-existing organ damage, either acute or chronic, before the infection outbreak: the higher the SOFA score, the more chances of mortality.

Another measurement termed for sepsis is known as qSOFA (quick SOFA). It does not demand laboratory tests and can be determined rapidly and frequently. The criteria for qSOFA are respiratory rate greater than or equal to 22/min, less than or equal to 100 mm Hg systolic blood pressure, and altered mentation. *Septic shock* is a subgroup of sepsis, and mortality increases due to circulatory and cellular metabolism anomalies. Septic shock can be detected by patients suffering from hypotension: mean arterial pressure is 65 mm Hg. They required vasopressor therapy to regulate adequate pressure of blood and serum lactate level less than two mmol/L. This condition led to an increase in the chances of mortality by 40%^1^.

In last two decades small nucleotide molecules called microRNAs (miRNAs) came to highlight due to robust findings by various research groups in disease pathogenesis and tumorigenesis. miRNAs are 22–24 nucleotide long and non-coding in nature, generally binds to sites complementary to the target mRNAs, especially the 3ʹ untranslated region (UTR) with some exceptions. miRNA binding causes mRNA degradation, which may or may not require the repression of translation^3^. miRNAs are associated with the argonaute family of proteins, and this association leads to the formation of a silencing complex, which works in pathway regulation.

The miRNAs plays crucial role in several biological functions, such as cell cycle, apoptosis, angiogenesis, metabolism, inflammation, immunity, proliferation, and various lung injuries^4,5^. MicroRNAs shows pleiotropy, therefore they have multiple gene (mRNA) targets and vice-versa. The target regions for many miRNAs may sometimes co-locate together at single gene, resulting in a synergistic repression of mRNA^6,7^. Scientists also reported several miRNAs which functions in a cell or organ-specific fashion. A particular type of miRNA can affect many interlinked biological pathways via multiple target sequences. Thereby playing critical role in disease conditions.

miRNAs act as an excellent candidate to be deemed a biomarker because they fulfill all the necessary criteria, such as sensitivity, specificity, and accessibility^8^. As reported by several experimental reports, they play an active role in sepsis as the specific miRNAs expression profiling determines the many miRNAs have upregulated in neonates with sepsis^9^. Additionally, some miRNAs are reported to help in the diagnosis of brain disease related to sepsis-associated encephalopathy^10^. Furthermore, they play a crucial role in the tumorigenesis process by altering the various molecular processes such as metastasis, cell cycle, and apoptosis. Iqbal et al. discussed several miRNAs that are upregulated or downregulated in the case of lung cancer^8,11^. Any alteration in a miRNA expression level may help us estimate and distinguish disease viz. in case of sepsis^12^.

Regulating the transcription of genes is handled by transcription factors (TFs) which are protein molecules. Mutual regulation between TFs and miRNAs in a firmly connected fashion gives rise to feed-back loops (FBLs) or feed-forward loops (FFLs) wherein a TF controls a miRNA, or a miRNA restrains a TF, or both of them co-regulate a joint target. FFLs can be classified into 3 types with respect to their master regulators: TF-FFL, miRNA-FFL, and composite FFL. TF (master regulator) controls its companion miRNA and their joint target within a TF-FFL, whereas in a miRNA-FFL, miRNA (master regulator) suppresses its partner TF and their joint target. TF-FFL and miRNA-FFL unite to obtain a composite FFL, where TF and miRNA control/suppress each other along with their joint target^13–16^.

In the present study, sepsis-associated mRNA expression profile datasets corresponding to varying age groups (i.e., adult, elder, and children) were extracted from National Center for Biotechnology Information (NCBI)-Gene Expression Omnibus (GEO). Differentially expressed genes (DEGs) were identified in all three age groups, followed by overlapping robust sepsis-specific DEGs occurring in all three age groups. Enrichment and protein–protein interaction (PPI) modular analyses resulted in a total of four genes. These genes were further subjected to 3-node miRNA FFL analysis to reveal key TF, miRNA, and gene having a possible role in sepsis initiation and further development.

## Results

### Microarray data collection and preprocessing

Considering the specified search criteria in the methods section, we obtained 141 expression profiles. However, the datasets with accession numbers GSE80496 and GSE67652 were selected since only these two had varying age group samples (GSE80496: Children and GSE67652: Adult and Elder). The patient samples from GSE67652 were bifurcated into two categories (i.e., six healthy adults versus six adult sepsis and six healthy elder versus six elder sepsis). Also, a total of 42 children samples (i.e. 21 healthy control versus 21 sepsis) were taken into consideration for GSE80496. Information on both these datasets is demonstrated in Supplementary Table S1, respectively. Scatterplots illustrating normalized gene expression of elder, adult, and children age groups concerning their control samples are demonstrated in Supplementary Fig. S1A–C.

### Identification of sepsis-specific DEGs

**Figure 1:**
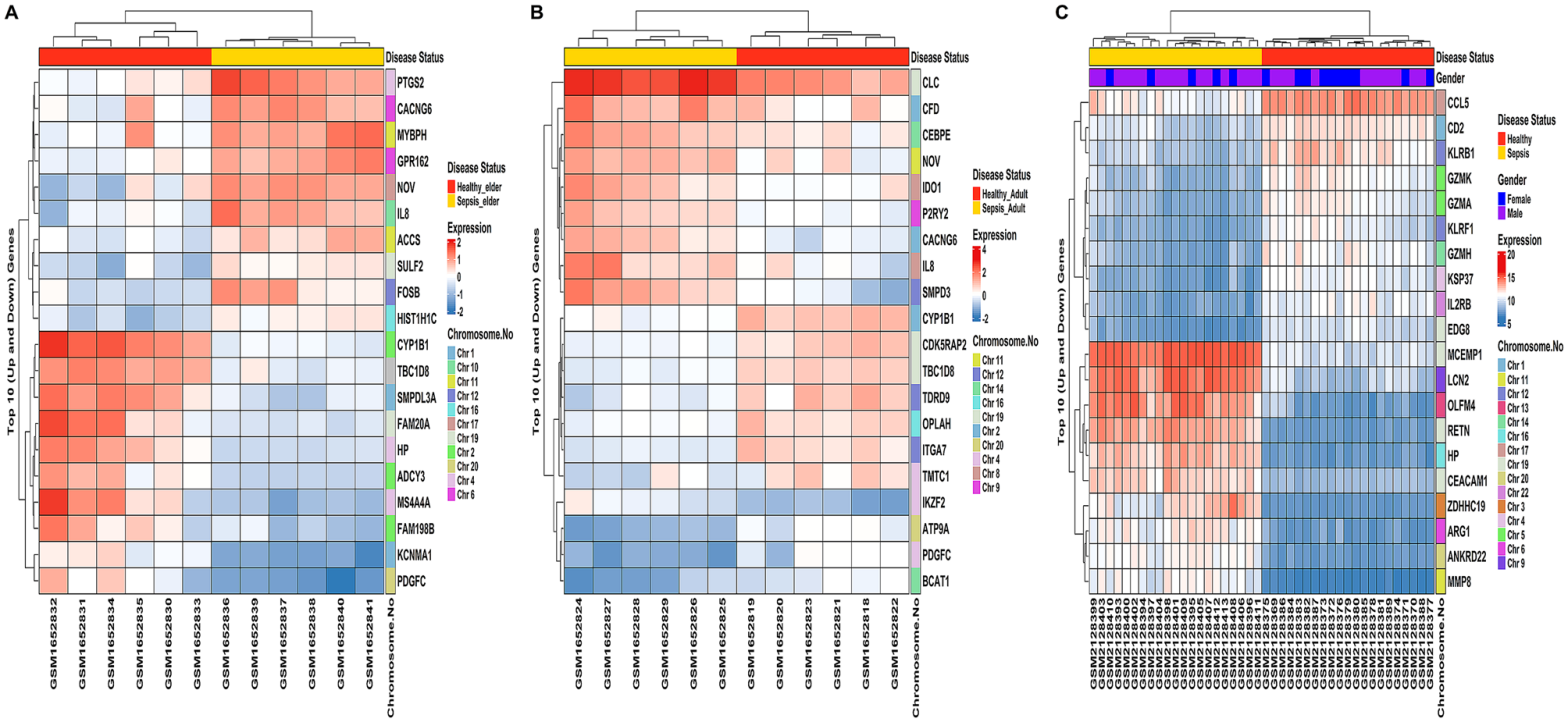
Heatmap of top 10 down and upregulated sepsis-specific DEGs in **(A)** elder (GSE67652), **(B)** adult (GSE67652), and **(C)** children (GSE80496) age groups. The expression of DEGs (within rows) is normalized across all the samples (within columns). The cluster dendrograms indicating hierarchical clustering (Euclidean distance-based) for both columns and rows are exhibited along the top and left sides of the plot. The gender type annotation bar (only for children age groups) and sample type annotation bar (for all age groups) are placed at the top of heatmaps. The position of genes on their respective chromosome number is shown as a vertical annotation bar (multicolor bands) in the right panel.

**Figure 2:**
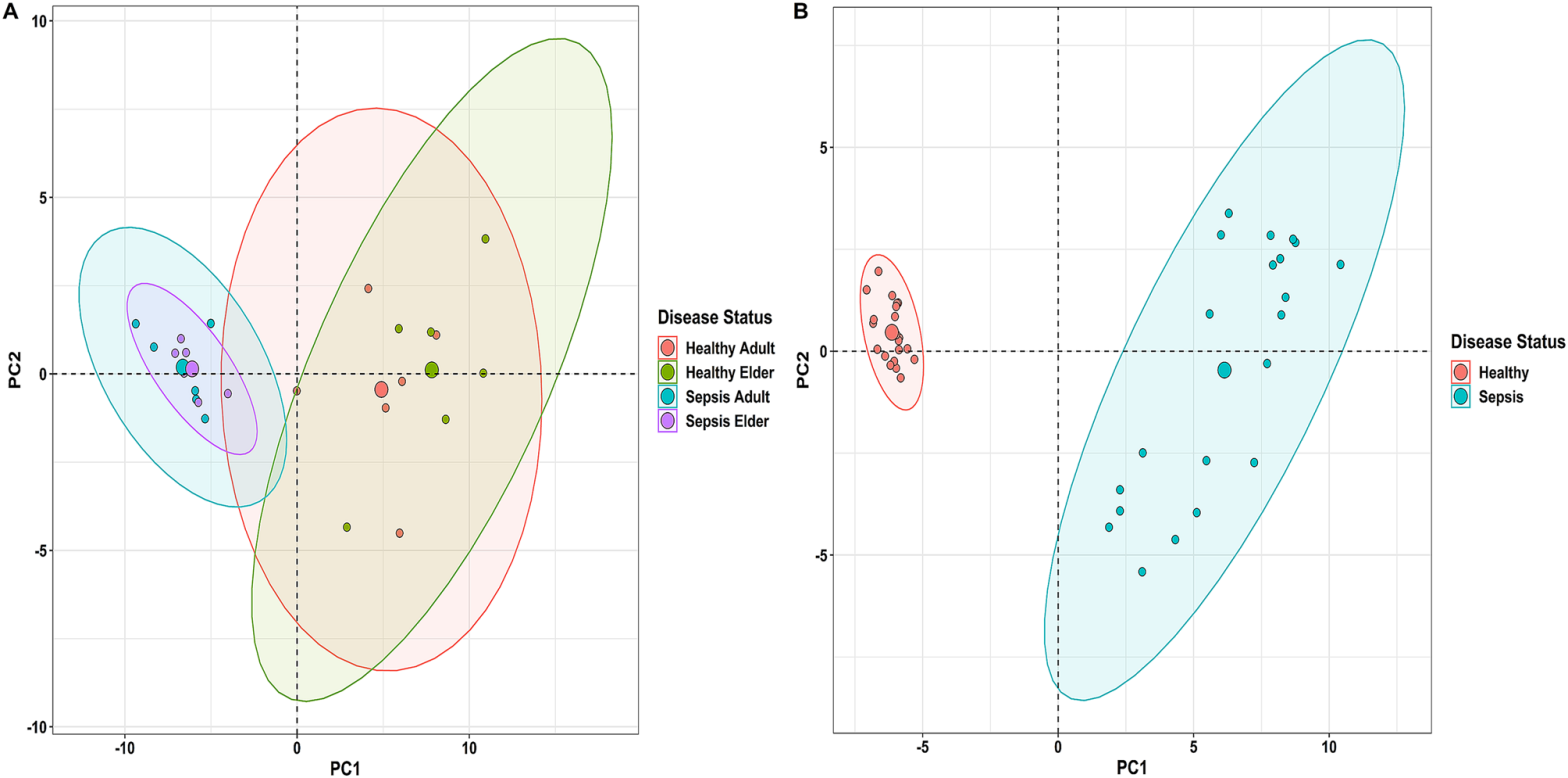
Principal component analysis (PCA) plots showing the expression variability of DEGs across all the samples in **(A)** GSE67652 and **(B)** GSE80496 datasets. Each point in the plot signifies the relative expression value of all DEGs dimensionally reduced concerning every sample leading to distinct cluster formations. Healthy adult, healthy elder, sepsis adult, and sepsis elder samples in (**A**) are represented by orange-, green-, cyan-, and magenta-colored points. The orange and cyan colored points represent healthy and sepsis samples in (**B**).

Following the abovementioned threshold [i.e., p-value $<0.05$ and $\left|{\text{log}}_{2}(\text{fold change})\right|>0.5$], 286, 153, and 2854 DEGs were identified in elder, adult, and children age groups against their respective control samples. The criterion for low fold change was incorporated in order to enlarge maximum number of DEGs between sepsis and healthy sample groups. Since there were very little number of genes differentially expressed at this fold change, making it more stringent would lead to nearly no DEGs or eliminating any important genes. The sums up of upregulated and downregulated DEGs in elder, adult, and children age groups are shown in Table 1. Supplementary Figure S2A–C demonstrates an overview of significant (up versus downregulated) and nonsignificant genes in elder, adult, and children age groups with volcano plots. Figure 1A–C shows heatmap plots of top 10 down and upregulated DEGs in elder, adult, and children age groups for their sample level. The sample type annotation bar is placed at the top of heatmaps. Figure 2A,B demonstrates principal component analysis (PCA) plots for GSE67652 and GSE80496 datasets. The expression variability of all DEGs in both datasets is dimensionally reduced, leading to distinct cluster formations with respect to each sample group. A total of 59 robust DEGs overlapped between elder, adult, and children age groups, as shown by the Venn plot in Supplementary Fig. S3.

**Table 1:** Total number of up and downregulated DEGs in all three age groups.

GEO accession	No. of upregulated DEGs	No. of downregulated DEGs
GSE80496 (Children)	1395	1459
GSE67652 (Elder)	131	155
GSE67652 (Adult)	75	78

### Pathway and Gene Ontology (GO) term enrichment analyses

**Figure 3:**
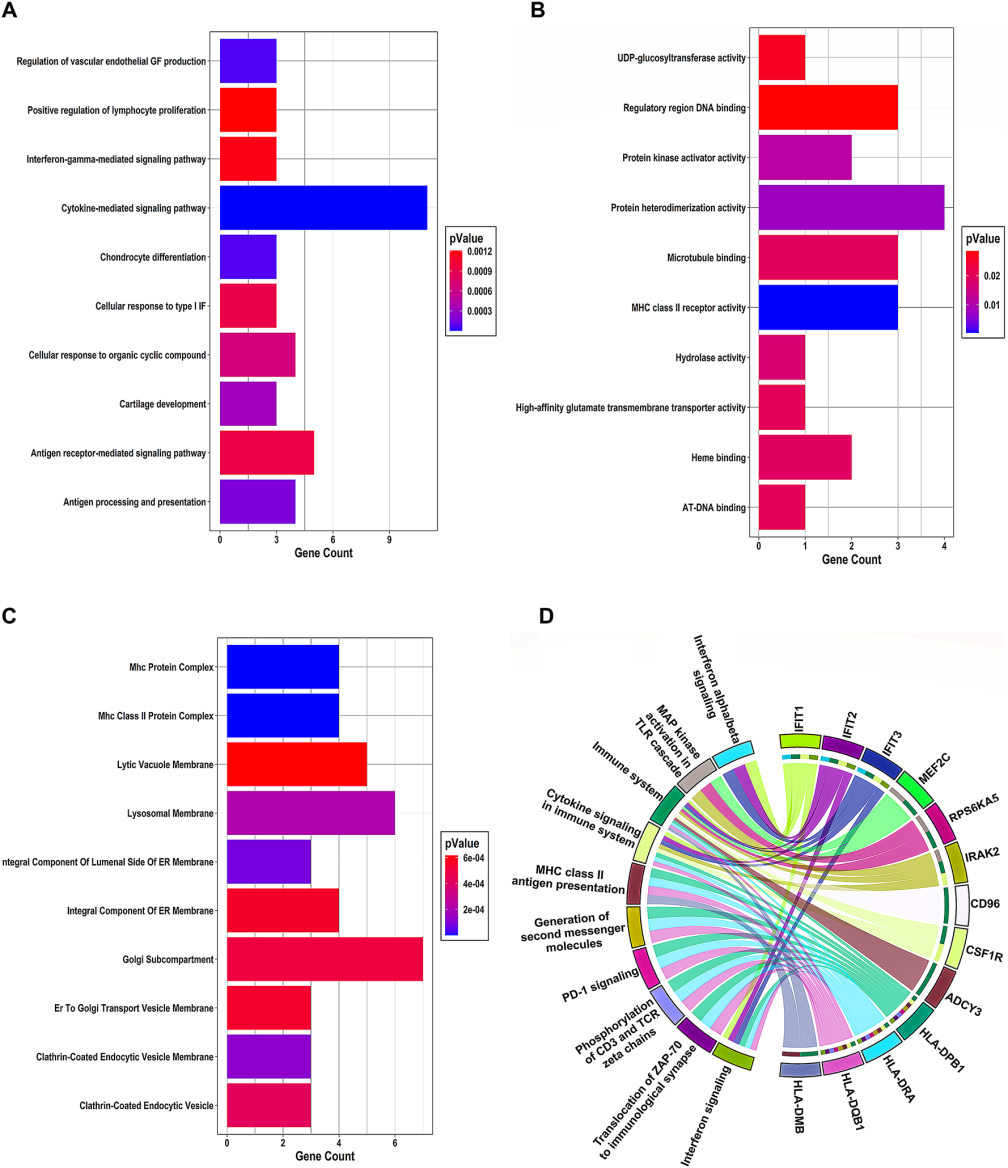
**(A)** Barplot offering significant GO-BP terms (on the y-axis) as bars concerning gene count (on the x-axis). The color of the bars varies following the p-value. **(B)** Barplot shows significant GO-MF terms (on the y-axis) as bars concerning gene count (on the x-axis). The color of the bars varies following the p-value. **(C)** Barplot showing the connection of significant GO-CC terms (on the y-axis) with corresponding gene count (on the x-axis). The bar color varies following the p-value. **(D)** Chord plot exhibiting the link of 13 DEGs (right semicircle) with ten significant pathways (left semicircle) via undirected colored edges.

A total of 19, 16, and 11 DEGs were engaged in the top 10 significant Biological Process (BP), Molecular Function (MF), and Cellular Compartment (CC) terms, respectively. Barplot of significant GO-BP terms (y-axis) concerning gene count (x-axis) is shown in Fig. 3A, where cytokine-mediated signaling pathway has the maximum number of genes (i.e., 11 genes). Barplot of significant GO-MF terms (y-axis) concerning gene count (x-axis) is demonstrated in Fig. 3B, where protein heterodimerization activity has the maximum number of genes (i.e., four genes). The association of significant GO-CC terms (y-axis) with corresponding gene count (x-axis) is demonstrated by a Barplot in Fig. 3C, with the Golgi subcompartment having the maximum number of genes (i.e., 7 genes). The most significant BP, MF, and CC terms were cytokine-mediated signaling pathway ($\text{p-value}={2.06 \times 10}^{-6}$), MHC class II receptor activity ($\text{p-value}={3.79 \times 10}^{-4}$), and MHC class II protein complex ($\text{p-value}={6.69 \times 10}^{-8}$). A total of 13 DEGs were involved in the top 10 significant pathways with Interferon signaling ($\text{p-value}={2.4 \times 10}^{-5}$) being the most significant pathway. Chord plot showing the link of these 13 DEGs with ten effective pathways is demonstrated in Fig. 3D. Linking edges in the chord plot display that HLA-DPB1, HLA-DQB1, and HLA-DRA were present in the highest number of pathways (i.e., eight pathways). Union of 34 pathway and functionally enriched DEGs were used for PPI construction and analysis.

### PPI network analysis and hub module detection

**Figure 4:**
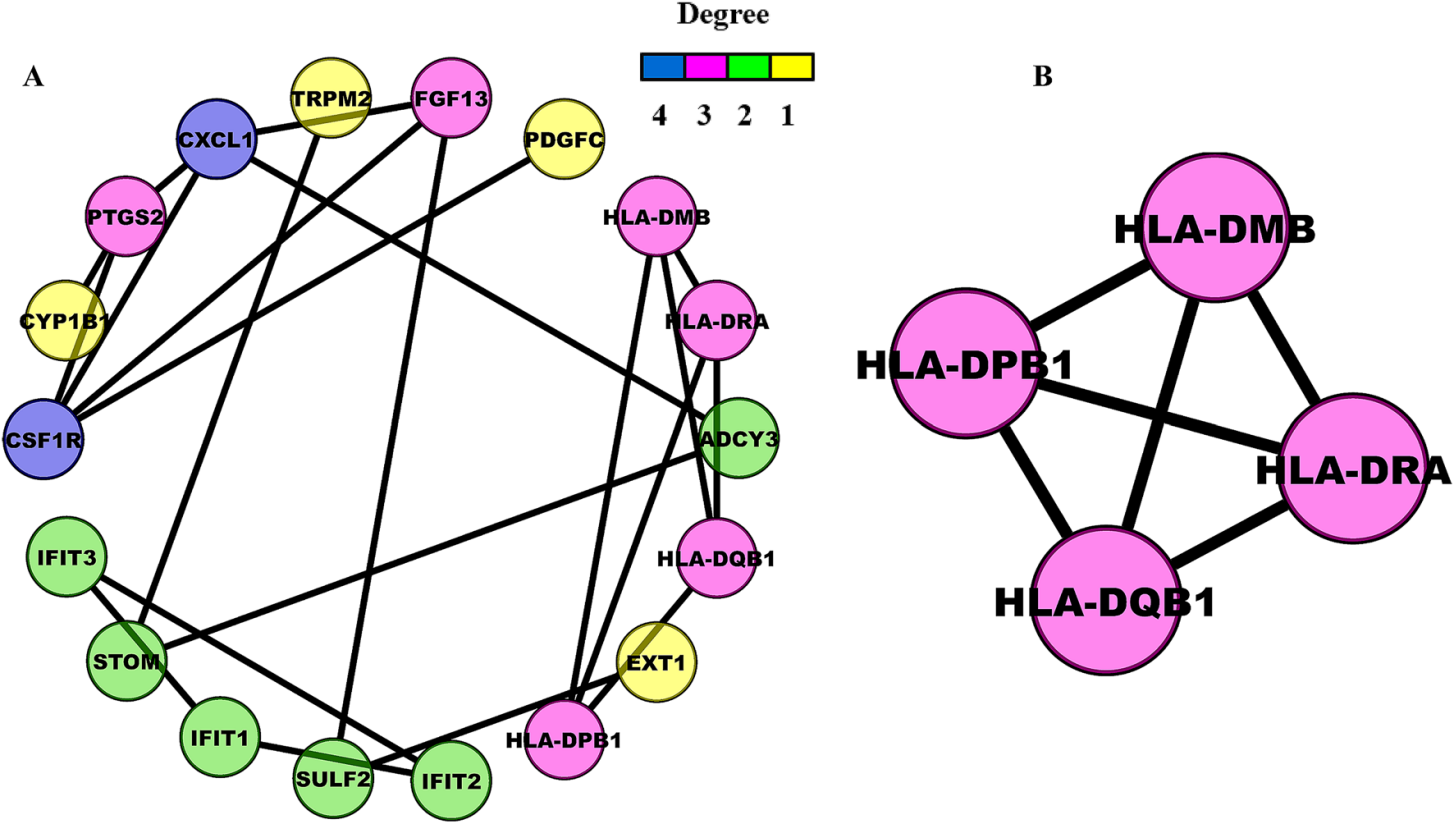
**(A)** Undirected PPI network containing 18 nodes and 21 edges corresponding to STRING interaction score $>0.4$. The node color varies with respect to degrees. **(B)** Highest-scoring PPI hub module with four nodes and six edges.

**Figure 5:**
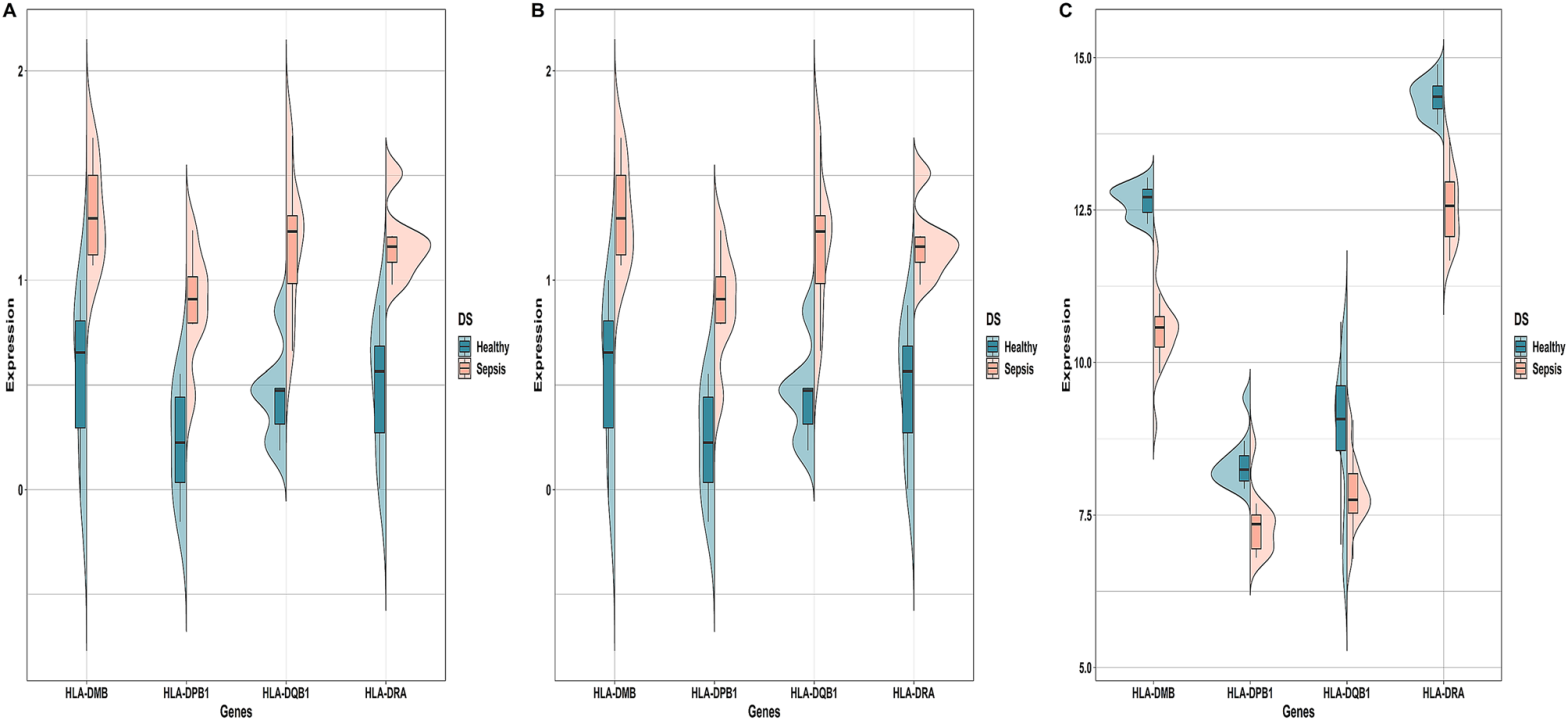
Split violin plots exhibiting expression intensity distribution of 4 hub DEGs (i.e., HLA-DPB1, HLA-DQB1, HLA-DRA, HLA-DMB) in **(A)** elder, **(B)** adult, and **(C)** children age groups. Dark green and brick red colors denote healthy and sepsis samples. The top and bottom of the boxes within the split violin depict the 75th and 25th percentile of the distribution. The horizontal lines within the boxes signify the median values. Axis endpoints are labeled by the minimum and maximum values, respectively.

18 out of 34 enriched DEGs were engaged in the PPI network corresponding to Search Tool for the Retrieval of Interacting Genes (STRING) interaction score $>0.4$. Figure 4A demonstrates that the PPI network comprises 18 nodes and 21 edges. Molecular Complex Detection (MCODE) revealed 3 PPI modules, out of which module-1 (score = 4) had the highest score and was considered the hub module. Figure 4B demonstrates the hub PPI module with four nodes and six edges. Essential centrality measures like node degree, betweenness, closeness, clustering coefficient, topological coefficient, and average shortest path length of PPI network are shown in Supplementary Fig. S4A–F. Split violin plots showing the expression distribution of 4 hub DEGs in elder, adult, and children age groups are demonstrated in Fig. 5A–C. Violin plots revealed that HLA-DQB1 and HLA-DMB were highly upregulated in the elder and adult age groups of the GSE67652 dataset. At the same time, HLA-DMB was highly downregulated in the GSE80496 dataset.

### Sepsis-specific 3-node miRNA FFL analysis

**Figure 6:**
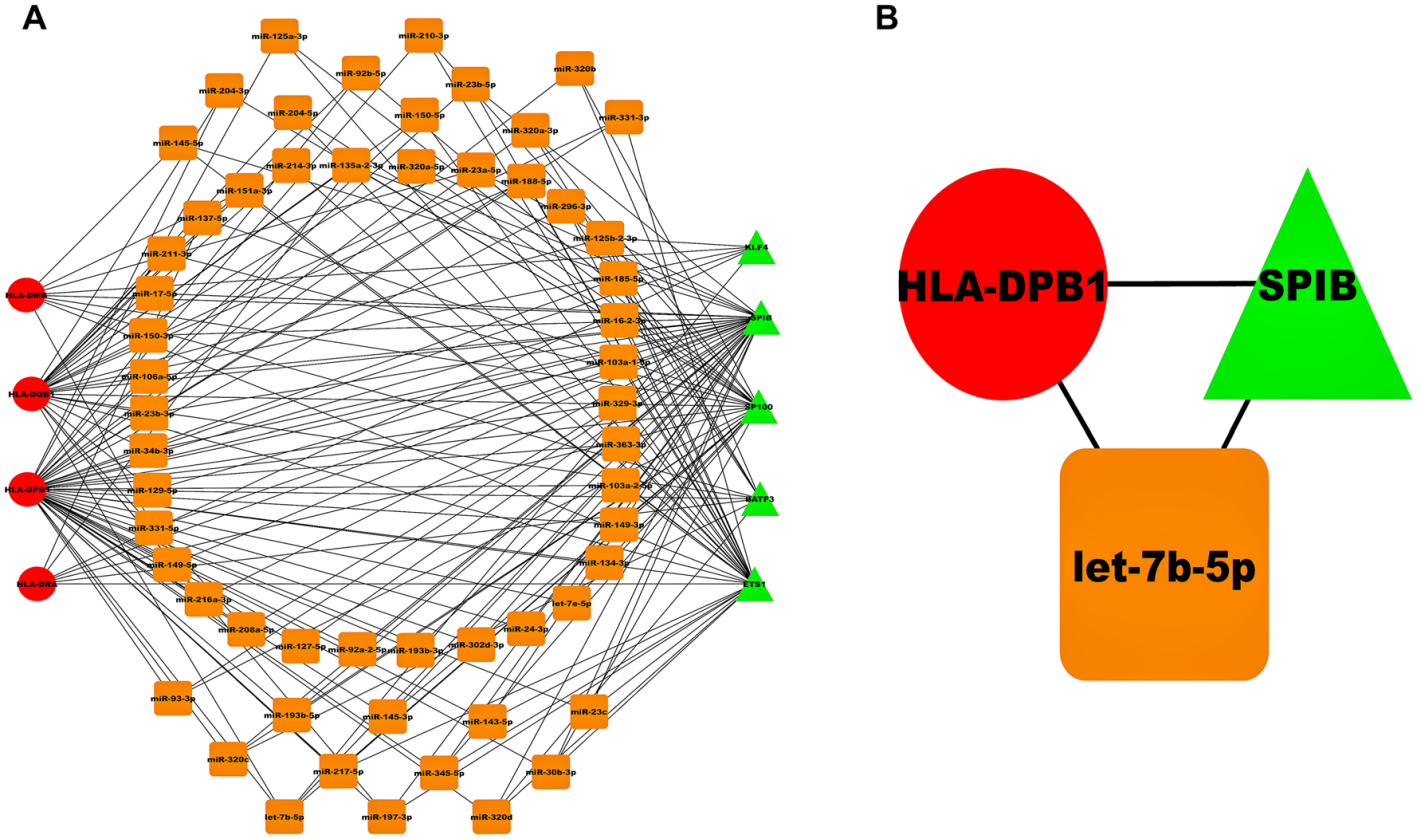
(**A**) Sepsis-specific 3-node miRNA FFL network with 66 nodes and 176 edges. (**B**) The highest-order FFL network motif comprises one hub TF (SPIB), one hub mRNA (HLA-DPB1), and one hub miRNA (let-7b-5p). Red-colored circular nodes signify hub DEGs, green-colored triangular nodes signify TFs, and golden-colored rectangular nodes signify miRNAs.

**Figure 7:**
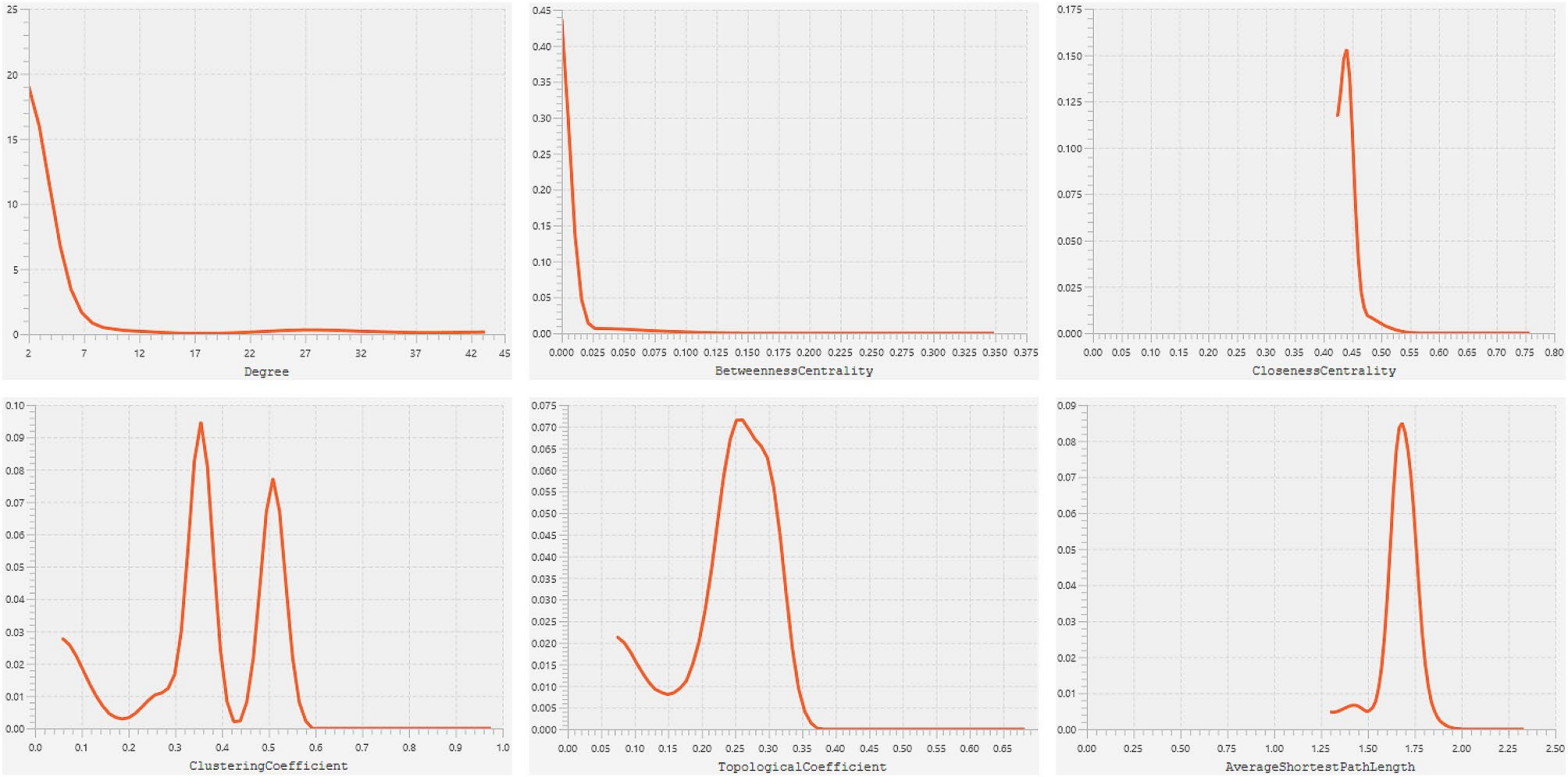
Centrality measures showing node degree distribution, betweenness, closeness, clustering coefficient, topological coefficient, and average shortest path length of miRNA FFL.

Our sepsis-specific 3-node miRNA FFL, as shown in Fig. 6A, comprises 66 nodes and 176 edges. Amongst all these edges, the TF-mRNA pair constituted 20 edges, whereas miRNA-TF and miRNA-mRNA pairs constituted 86 and 69 edges. Amongst total nodes, 5, 4, and 57 belonged to TFs, mRNAs, and miRNAs. Within this network, degree values of miRNAs and mRNAs varied from 2 to 4 and 7 to 45. The average degrees of mRNAs and miRNAs were 22.25 and 2.71. Supplementary Table S2 summarizes all the three regulatory relationships between TFs, miRNAs, and mRNAs. Out of all TFs, SPIB was repressed by the maximum miRNAs (i.e., 31). Moreover, among all our hub DEGs, HLA-DPB1 was suppressed by the maximum miRNAs (i.e., 40). Supplementary Table S3 shows miRNA FFL nodes ranked based on betweenness, degree, closeness, and radiality. The table shows that let-7b-5p, SPIB, and HLA-DPB1 were the highest-ranked miRNA, TF, and mRNA based on these centralities. Thus, these elements act as hubs that might play an essential role in sepsis. Based on all these observations, the highest-order subnetwork motif comprises one TF (SPIB), one miRNA (let-7b-5p), and one mRNA (HLA-DPB1), as shown in Fig. 6B. Essential centrality measures like node degree, betweenness, closeness, clustering coefficient, topological coefficient, and average shortest path length of miRNA FFL are demonstrated in Fig. 7. List of top 10 significant (p-value $< 0.05$) GO terms associated with let-7b-5p is shown in Supplementary Table S4 where Hepatic Stellate Cell Differentiation (p-value = $2.85\times {10}^{-3}$) was the most significant amongst all. Also, the list of all signaling pathways associated with let-7b-5p is shown in Supplementary Table S5.

## Discussion

Highly advanced technology such as microarray serves the information by analyzing genes involved in medical conditions. We used GEO database to determine the two datasets, GSE80496 (n = 24) and GSE67652 (n = 42). These two datasets contain the genes expressed in the microarray profile of the healthy and sepsis population. We found out that the DEGs from the two datasets of the healthy population and sepsis population of children, adults, and elders. A total of 1601 upregulated DEGs and 1692 downregulated DEGs were found in these two datasets. However, a total of 59 common sepsis-linked DEGs were introduced among the population of children, adults, and elders suffering from sepsis. Sepsis in infants and children diverge in its etiology, pathophysiology, diagnosis and management than adults. The congenital heart disease and chronic lung disease are comorbid conditions which is found in infants with sepsis. The spot of infection is also dependent of age, Prusakowski et al. reported a higher risk of morbidity and mortality in infants than young children in case of sepsis. We may hypothesize that more DEGs in children among three age groups is due to more pathophysiological changes^17^.

The GO:BP analysis provided the list of possible PPIs or co-regulation associations with the following pathways cytokine-mediated signaling pathway, the pathway of antigen receptor-mediated signaling, presentation of exogenous peptide antigen via MHC class II and antigen processing, the path of interferon-gamma-mediated signaling, and proliferation of positive regulation of lymphocyte. Node degree, betweenness, closeness, and stress help analyze the significant connections in biological networks. GO:CC find out following terms related to HLA-DPB1, MHC protein complex, clathrin-coated endocytic vesicle membrane, an integral member of lumenal side of the ER membrane, MHC class II protein complex, lysosomal membrane, clathrin-coated endocytic vesicle, Golgi subcompartment, an essential part of endoplasmic reticulum membrane, lytic vacuole membrane, and COPII-coated ER to Golgi transport vesicle. DEGs were evaluated from the PPI network by examining the critical network centrality assessment.

Pathway and GO term mesmerize the genes that are affected in sepsis. PPI was made to decode the hidden molecular mechanism in the disorganized physiological processes linked with sepsis. A collection of 18 significant pathway and functionally embellished DEGs have been analyzed for PPI construction and hub module identification. From these networks, four genes were identified based on their centrality assessment. The upregulated genes are HLA-DQB1 and HLA-DMB in the elder and adult age groups of the GSE67652 dataset.

In comparison, HLA-DMB was highly downregulated in the GSE80496 dataset. Our study estimated a regulatory network-based proposal to detect specific DEGs, miRNAs, and TFs that form sepsis-specific closed 3-node miRNA FFL network. Analyzing these FFL may help understand the cell and molecular pathway dysregulated in sepsis, thus encoding its pathophysiology. The outcome of our study detected one miRNA, i.e., miR-let-7b-5p, which regulates the HLA-DPB1 and SPIB.

Human Leukocyte Antigen (HLA) complex is a set of genes present on chromosome number 6; it plays a major role in immune system functioning. HLA-DPB is a paralogue of the HLA class II beta chains. HLA-DBP is a heterodimer comprised of single alpha and a single beta chain anchored in the cell membrane. HLA-DBP works by presenting peptides of extracellular proteins to other immune cells, thus playing a crucial role in the body's immune system. Experimental results of one research group showed that HLA class second alleles could play a vital role in smallpox vaccine-induced adaptive immunity. These results implicate the DPB1 function in adaptive immune response; therefore, further studies can significantly associate HLA-DPB1 in the adaptive immune response to sepsis^18^. A research study on primary human acute myeloid leukemia showed that HLA-DBP1 specific TCRs genetic transfer into CD4+ and CD8+ T cells could provide an enhanced immune reaction to AML blasts via cytolytic activity and IFN-gamma production in the AML xenograft mouse model^19^.

Pathways are closely associated with HLA-DPB1, namely Interferon Signaling, Phosphorylation of CD3 and TCR zeta chains, translocation of ZAP-70 to Immunological synapse, PD-1 signaling, MHC class II antigen presentation, Generation of second messenger molecules, and Cytokine Signaling in Immune system but further experimental studies needed to gain robust insights of DBP1 association.

Post-operative abdominal infections are identified as usual triggers of sepsis worldwide. Siegler and the group designed an experiment to study sepsis-induced CCCTC-Binding Factor (CTCF) differential occupancy in post-operative sepsis patients and its contribution to altering the transcriptional response of human monocytes during illness. They find a selective incrementation in CTCF binding inside MHC-II. Consequently, transcriptional decreases in HLA-DPA1, HLA-DPB1, HLA-DRB1, and HLA-DRA. In conclusion, their study reveals that during post-operative sepsis in humans, there is CTCF involvement in the modulation of the transcriptional response of functional monocytes^20^.

The DEGs established using computational analysis are overall managed by TFs and miRNAs. TFs are involved in transcribing DNA into RNA, cis-regulating molecules that initiate and regulate transcription of genes and preset on the gene’s promoter region.

Primary miRNAs and TFs linked with sepsis-linked DEGs are classified via a FFL. Spi-B, also known as SPIB, is a PU-box binding transcriptional activator that binds to the specific purine-rich DNA sequence^21^. Experimental results showed that the binding of TF SPIB to DNA sequence may function as a lymphoid-specific enhancer. Moreover, SPIB promoted plasmacytoid dendritic cells (pDCs) development^22^. pDCs are known to be an interferon (IFN) producer. Some studies also demonstrated SPIB requirement in BCR signaling, in turn, required for B-cell development and antigenic stimulation^23^.

Our finding identified miR-let-7b-5p in FFL modulation, which associates SPIB and HLA-DPB1 in sepsis. Bioinformatics and experimental data suggest miR-let-7b role in DNA repair and found differential expression of miR-let-7b in breast cancer^24^. Likewise, there is prominent downregulation of miR-let-7b in osteosarcoma cell lines and tissues, and results conveyed its antitumor effects via interaction with IGF1R and inhibiting IGF1R expression^25^. Several shreds of evidence also suggested miR-let-7b function in inflammation by targeting PI3K, AKT, HMGA2, and SIRT1 genes. Differential gene expression studies also revealed let-7b as DEG in inflamed tissue samples and normal tissue. miR-let-7b overexpression led to a significant decrease in pro-inflammatory gene expression and vice versa. With the help of other circulating microRNAs and non-coding RNAs, let-7b acts as a regulator of inflammation. One published study demonstrated that let-7b regulates macrophage polarization in prostatic tumors and modulates prostate cancer prognosis^26^.

Islakoglu et al. found out the meta-miRNA lists involved in diseases through the miRCancer and PhenomiR 2.0; after that, they found that nine miRNAs were prevalent. When pathway analysis is executed with these miRNAs’ targets, they are remarkably embellished in critical pathways in cancer, such as p53 and PI3K-Akt signaling pathways. They found out that let-7b-5p and the other three members of the let-7 family were important in the Estrogen receptor (ER) pathological subtype of breast cancer. Barh et al. reported that let-7 family members might govern the angiogenic pathway and ER signaling and regulate apoptosis and cancer stem-cell differentiation^27,28^.

Additionally, in the case of H. pylori, infection let-7b can help in epithelial immune response^29^. Studies also revealed that miR-let-7b could prevent autophagy and apoptosis of mesenchymal stem cells implanted in infarcted myocardium^30^. Furthermore, let-7b may be a potential biomarker for multiple sclerosis^31^. Cheng Gao et al. reported that let-7b-5p expression was lower in kidney tissues of sepsis patients than healthy ones. They also found that LPS can also inhibit the expression of let-7b-5p in time and dose-dependent manners. LPS induced cell death can be partially reduced by upregulation of let-7b-5p miRNA in the HK-2 cell line. They have observed that transfection of let-7b-5p into HK-2 cells decreased the expression of IL-6, TNF-α, and IL-1β after LPS-induced inflammation^32^. Previous experimental studies suggested the role of miR-let-7b in suppressing tumors, which works by inhibiting tumor proliferation, invasion, and adhesion via interacting with various genes named HMGA2, RDX, DIAPH2, Ras c-myc, and PKA1^33^. let-7b-5p also downregulated the expression level of Suppressor of cytokine signaling 1 (SOCS1) and hiked the phosphorylation of STAT1, STAT5a, and STAT3 proteins in prostatic macrophages. It may also regulate the M1/M2 polarization by SOCS1/STAT pathway^34^. This lays a strong emphasis on the miRNA–mRNA regulatory network. This regulatory network can support our understanding of the disease and help us determine the innovative therapeutic aspects to help recover patients suffering from sepsis and different lung ailments.

## Conclusions

However, in vitro and in vivo studies are obligate to establish the role of the identified gene in the pathogenesis and therapeutic strategies of sepsis.

## Materials and methods

### Microarray data collection and preprocessing

NCBI-GEO (https://www.ncbi.nlm.nih.gov/geo/)^35^ was accessed to retrieve sepsis-associated mRNA expression profiles with “sepsis” used as a suitable keyword during searching. The search results were further trimmed down by applying inclusion criteria as follows: (1) all samples must belong to “Homo Sapiens” and “expression profiling by array” type; (2) the dataset(s) must be having both healthy control and sepsis samples; (3) the dataset(s) must be having diverse age group samples (i.e., adult, elder, children); (4) the dataset(s) must be having processed and raw microarray data; (5) the dataset(s) must be submitted within last 10 years (i.e. 2011–2021); (6) the dataset(s) must be having patient samples in the range of 20 to 1000. Case reports, abstracts, cell-line based experimental study designs, review articles, and studies without non-human samples or healthy controls were excluded. The series matrix expression files of selected dataset(s) were downloaded using the GEOquery R package^36^. Probe ID to HUGO Gene Nomenclature Committee (HGNC) symbols mapping was implemented utilizing feature data stored in the expression set object of respective datasets. The expression values were averaged for those gene symbols that mapped to more than one probe IDs in order to avoid redundancy.

### Identification of sepsis-specific DEGs

DEGs between normal and age-related sepsis samples were identified using a two-sample t-test for individual datasets. The p-values and ${\text{log}}_{2}(\text{fold change})$ values for all the genes in individual datasets across two samples were computed using the Limma R package^37^ followed by p-value correction using Benjamini-Hochberg (BH) method^38^. Genes corresponding to a p-value < 0.05 and $\left|{\text{log}}_{2}(\text{fold change})\right|>0.5$ were considered as differentially expressed across two sample types. DEGs with ${\text{log}}_{2}\left(\text{fold change}\right)>0.5$ and ${\text{log}}_{2}\left(\text{fold change}\right)<- 0.5$ were categorized as up and downregulated, respectively. Overlapping DEGs concerning varying age-related samples from individual datasets were considered highly robust and used for further analysis.

### Pathway and GO term enrichment analyses

Pathway and GO term enrichment data for overlapping age-spectrum sepsis DEGs were compiled using the ReactomePA package^39^ and GO-BP, MF, and CC libraries in the Enrichr database^40^. GO terms and pathways corresponding to a p-value $<0.05$ were statistically significant.

### PPI network construction and hub module detection

Union of significant pathway and functionally enriched DEGs were inputted to the STRING, (https://string-db.org/) v11.0 web-based tool^41^ for the construction of PPI network. The PPI network was formed at medium confidence (corresponding to interaction score $>0.4$) and afterwards visualized using Cytoscape v3.9.0^42^. The MCODE Cytoscape app^43^ was used to identify densely correlated modules within our PPI network. Default MCODE parameters (i.e., “Degree cutoff = 2”, “node score cutoff = 0.2”, “k-score = 2”, “max. depth = 100”, and “cut style = haircut”) were used for network scoring, and module detection and highest-scoring module was chosen as the hub module.

### Sepsis-specific 3-node miRNA FFL construction and analysis

Significant human TFs corresponding to score (p-value) $<0.05$ and regulating our hub mRNAs were fetched from the ChEA v3.0 database^44^. Then, miRNAs (with a score $> 0.95$ and binding only on ${3}^{{{\prime}}}\text{ UTR}$ region) repressing our hub mRNAs and extracted significant TFs (collected from ChEA) were extracted from miRWalk v3.0 database^45^. These miRNAs were then validated using literature, and those associated with sepsis were retained. To form a 3-node miRNA FFL, only common miRNAs repressing our mRNAs and TFs were retained. Lastly, all the three interaction pairs (i.e., miRNA-mRNA, TF-mRNA, and mRNA-TF) were altered concerning common miRNAs and final TFs. These pairs were then fused to obtain 3-node miRNA FFL^16^ and analyzed using Cytoscape. TAM v2.0 (http://www.lirmed.com/tam2/) and miR + Pathway (http://www.insect-genome.com/miR-pathway/Enrichment_analysis) were utilized for predicting GO terms and pathways corresponding to our key miRNA (within 3-node miRNA FFL).

## Supplementary Information

Supplementary Information 1.

Supplementary Information 2.

## Data Availability

The datasets used in our study were already available from Gene Expression Omnibus (GEO) under accession numbers GSE80496 (https://www.ncbi.nlm.nih.gov/geo/query/acc.cgi?acc=GSE80496) and GSE67652 (https://www.ncbi.nlm.nih.gov/geo/query/acc.cgi?acc=GSE67652).
