# Supplementary material for: Integrated transcriptomic and regulatory network analyses uncovers the role of let-7b-5p, SPIB, and HLA-DPB1 in sepsis
Source: Sci Rep. 2022 Jul 13;12:11963. doi: 10.1038/s41598-022-16183-6 (PMC9279366; doi:10.1038/s41598-022-16183-6)
Supplement: Supplementary file 2 — Supplementary Information 2. [file 41598_2022_16183_MOESM2_ESM.docx]

**Integrated transcriptomic and regulatory network analyses uncovers the role of let-7b-5p, SPIB, and HLA-DPB1 in Sepsis**

Mohd Mohsin^1,ϯ^, Prithvi Singh^2,ϯ^, Salman Khan^1^, Amit Kumar Verma^1^, Rishabh Jha^2^, Mohammed A. Alsahli^3^, Arshad Husain Rahmani^3^, Saleh A. Almatroodi^3^, Faris Alrumaihi^3^, Nisha Kaprwan^4^, Kapil Dev^1^, Ravins Dohare^2,*^ and Mansoor Ali Syed^1,*^

**^1^** Department of Biotechnology, Faculty of Natural Sciences, Jamia Millia Islamia, New Delhi 110025, India

**^2^** Centre for Interdisciplinary Research in Basic Sciences, Jamia Millia Islamia, New Delhi 110025, India

**^3^** Department of Medical Laboratories, College of Applied Medical Sciences, Qassim University, Buraydah 51452, Saudi Arabia

**^4^** Department of Computer Science, Faculty of Natural Sciences, Jamia Millia Islamia, New Delhi 110025, India

**^Ϯ^** These authors contributed to this work as first authors.

**^*^Correspondance:**

**Mansoor Ali Syed**, PhD **Ravins Dohare**, PhD

Assistant Professor Assistant Professor

Department of Biotechnology Centre for Interdisciplinary Research in Basic Sciences

Jamia Millia Islamia, New Delhi-110025 Jamia Millia Islamia, New Delhi-110025

**E-mail:** [smansoor@jmi.ac.in](mailto:smansoor@jmi.ac.in) **E-mail:** [ravinsdohare@gmail.com](file:///D:\BIOINFORMATICS\SESPSIS%20FINAL\ravinsdohare@gmail.com)

**Tel:** +91-9953786440 **Tel:** +91-9868655958


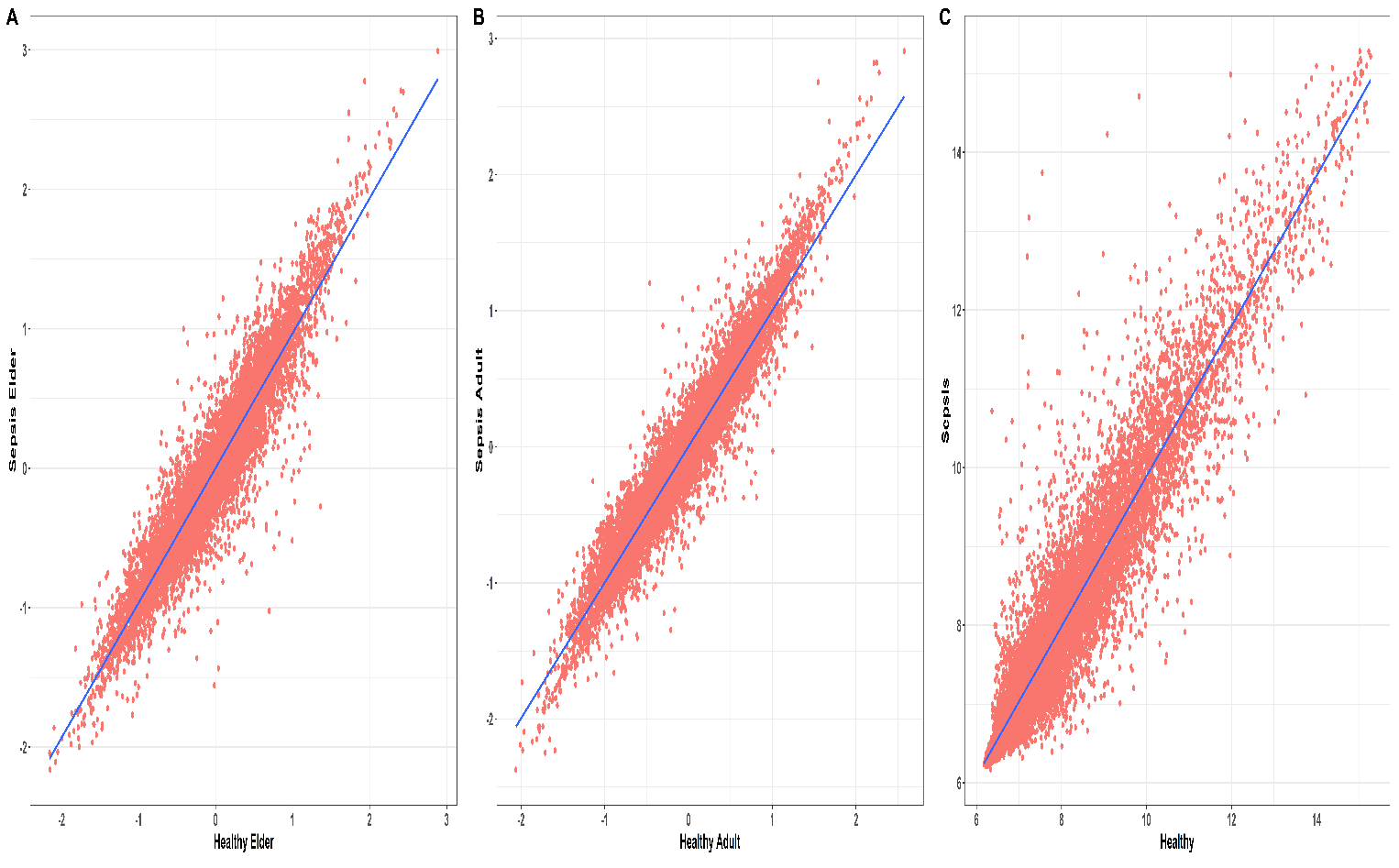


***Figure S1.*** *Scatterplots showing normalized gene expression between controls and* ***(A)*** *elder,* ***(B)*** *adult,* ***(C)*** *children sepsis age group samples. These plots were constructed using the mean values for each sepsis age group and their corresponding controls.*


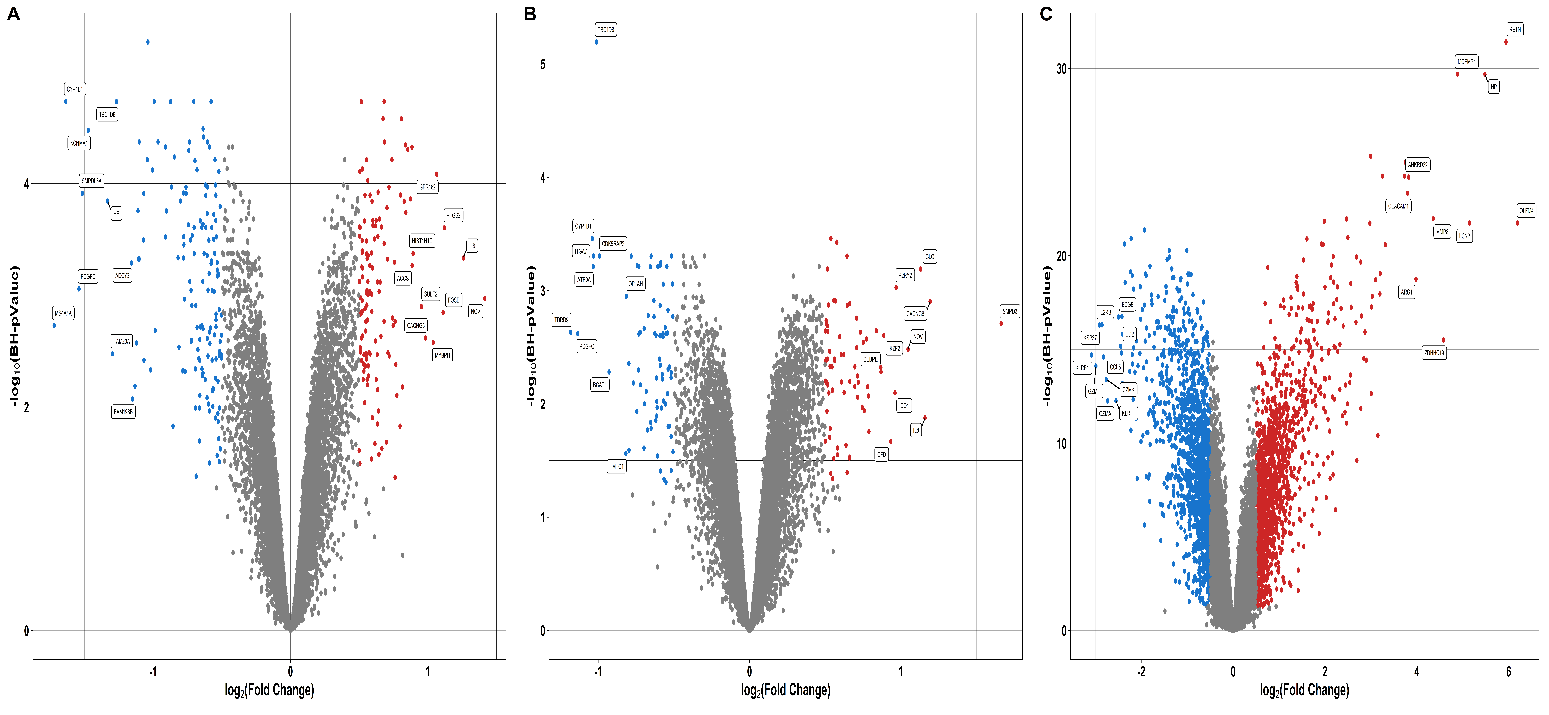


***Figure S2.*** *Volcano plots highlighting* ***(A)****286,* ***(B)****153, and* ***(C)*** *2854 DEGs against nonsignificant genes in elder, adult, and children age groups, respectively. The red and blue colored points signify up and downregulated DEGs. Gray colored points signify nonsignificant genes. The x and y axes represent the* ${log}_{2}\left( fold change \right)$ *and* ${-log}_{10}\left( BH-p-value \right)$*.*


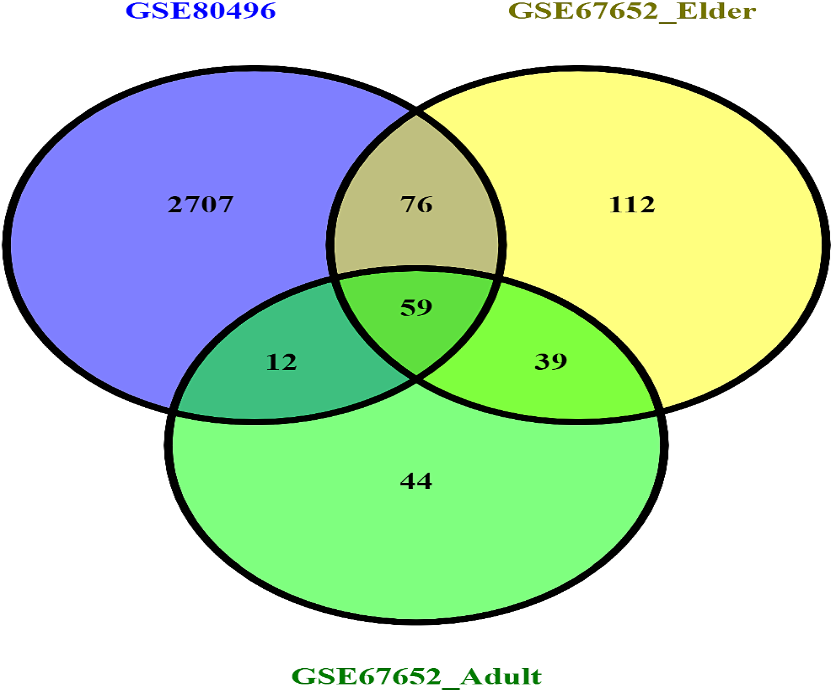


***Figure S3.*** *Venn plot showing the overlapping DEGs (59) between elder, adult, and children age groups. Blue, yellow, and green color filled circles denotes children, elder, and adult DEG sets.*

**
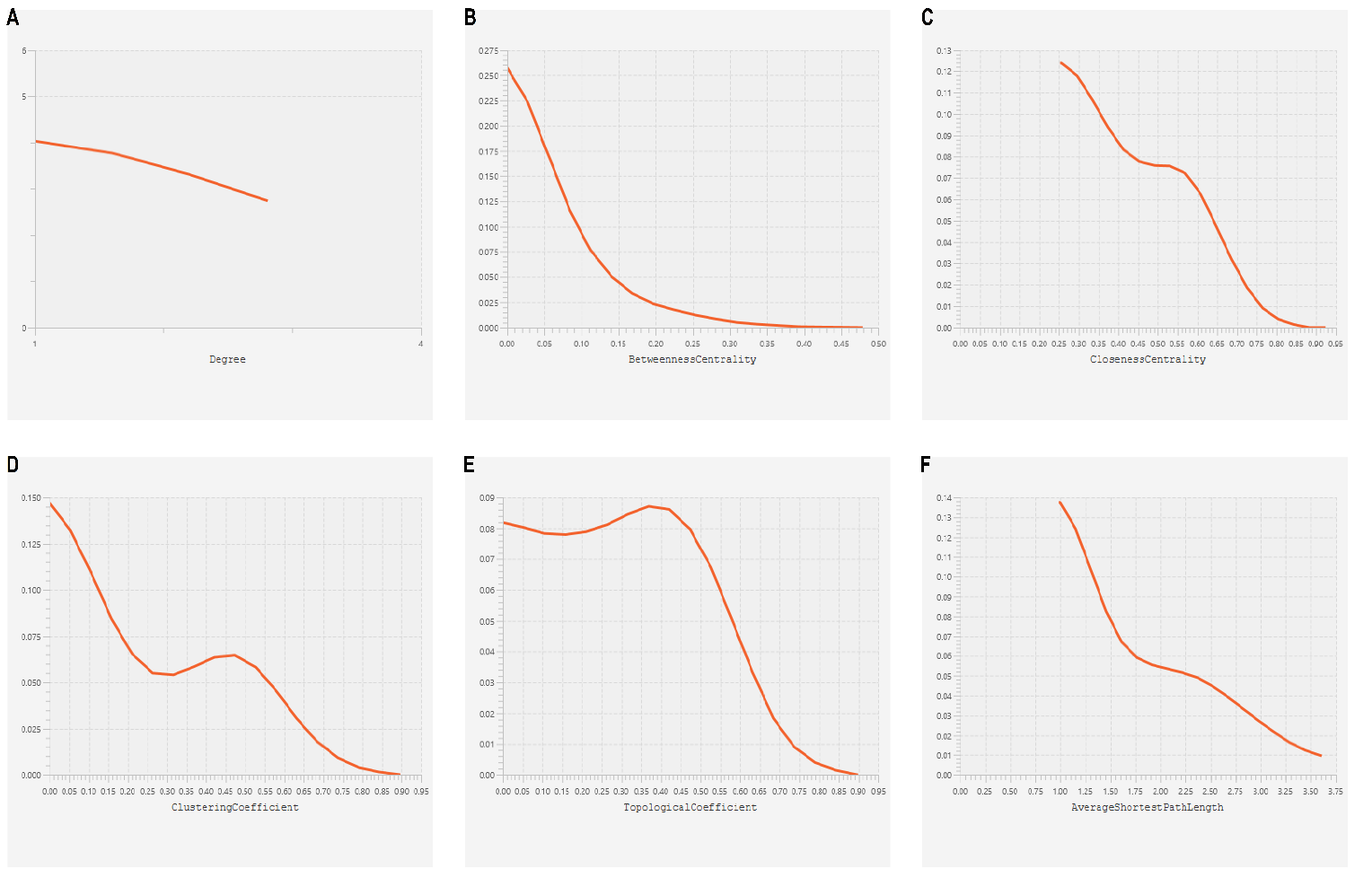
*Figure S4.*** *Centrality measures showing* ***(A)*** *node degree distribution,* ***(B)*** *betweenness,* ***(C)*** *closeness,* ***(D)*** *clustering coefficient,* ***(E)*** *topological coefficient, and* ***(F)*** *average shortest path length of PPI network.*

|  | Dataset 1 | Dataset 2 |
| --- | --- | --- |
| GEO Accession No. | GSE67652 | GSE80496 |
| Type of study | Expression profiling by array | Expression profiling by array |
| Platform type | Agilent-039494 SurePrint G3 Human GE v2 8x60K Microarray 039381 | Illumina HumanRef-8 v3.0 expression beadchip |
| Species | Homo sapiens | Homo sapiens |
| No. of samples | 24 (Healthy adult = 6, Sepsis adult = 6, Healthy elder = 6, Sepsis elder = 6) | 42 (Healthy = 21, Sepsis = 21) |

**Table S1.** Characteristics of sepsis-associated datasets retrieved from GEO

| Relationship | No. of edges | No. of miRNAs | No. of TFs | No. of mRNAs |
| --- | --- | --- | --- | --- |
| miRNA-mRNA^a^ | 69 | 57 |  | 4 |
| TF-mRNA^b^ | 20 |  | 5 | 4 |
| miRNA-TF^c^ | 86 | 57 | 5 |  |

**Table S2.** Summary of regulatory relationships between sepsis-associated mRNAs, miRNAs, and TFs

**^a^**miRNA-mRNA: miRNA repression of genes; **^b^**TF-mRNA: TF regulation of genes; **^c^**miRNA-TF: miRNA repression of TFs.

| GO Term | p-value | Fold |
| --- | --- | --- |
| Hepatic Stellate Cell Differentiation | ${2.85\times10}^{-3}$ | 351.2 |
| Anti-Cell Proliferation | ${4.56\times10}^{-3}$ | 219.5 |
| Folliculogenesis | ${6.26\times10}^{-3}$ | 159.6 |
| T-Cell Differentiation | ${9.11\times10}^{-3}$ | 109.7 |
| Cell Division | ${9.68\times10}^{-3}$ | 103.2 |
| Pluripotent Stem Cells Reprogramming | 0.011 | 87.8 |
| Glucose Metabolism | 0.015 | 62.7 |
| Bone Regeneration | 0.017 | 58.5 |
| Adipocyte Differentiation | 0.023 | 42.8 |
| Innate Immunity | 0.023 | 41.8 |

**Table S4.** List of significant GO terms (function) associated with hsa-let-7b-5p obtained from TAM 2.0 database

**Table S5.** List of signaling pathways associated with hsa-let-7b-5p obtained from miR+Pathway database.

| Signaling Pathways associated with hsa-let-7b-5p | |
| --- | --- |
| Adipocytokine signaling pathway | ErbB signaling pathway |
| AMPK signaling pathway | Estrogen signaling pathway |
| cGMP-PKG signaling pathway | Fc epsilon RI signaling pathway |
| MAPK signaling pathway | Relaxin signaling pathway |
| Wnt signaling pathway | Prolactin signaling pathway |
| Jak-STAT signaling pathway | PI3K-Akt signaling pathway |
| TGF-β signaling pathway | FoxO signaling pathway |
| Sphingolipid signaling pathway | Oxytocin signaling pathway |
| T cell receptor signaling pathway | Insulin signaling pathway |
| Hippo signaling pathway | Oxytocin signaling pathway |
| Calcium signaling pathway | AGE-RAGE signaling pathway |
| Apelin signaling pathway | Phospholipase D signaling pathway |
| cAMP signaling pathway | RIG-I-like receptor signaling pathway |
| Chemokine signaling pathway | Ras signaling pathway |
| VEGF signaling pathway | Rap1 signaling pathway |
| P53 signaling pathway | Hedgehog signaling pathway |
| B cell receptor signaling pathway | Glucagon signaling pathway |
| Thyroid hormone signaling pathway | GnRH signaling pathway |
| TNF signaling pathway | mTOR signaling pathway |
| Toll-like receptor signaling pathway | Neurotrophin signaling pathway |
| C-type lectin receptor signaling pathway | NOD-like receptor signaling pathway |
| Ras signaling pathway | NF-k β signaling pathway |
